# A Medical Arabic Course for Healthcare Professionals

**DOI:** 10.1007/s10903-025-01793-9

**Published:** 2025-10-25

**Authors:** Salma Fleifil, Lanah Almatroud, Jad Fawaz, Mohammad Turaani, Cindy Hakim, Lena Juratli, Reem Farjo, Hanin Elhagehassan, Ibrahim Khalaylih, Shahzad Mian, Emad Abou-Arab

**Affiliations:** 1 University of Michigan–Ann Arbor, Ann Arbor, United States; 2 Michigan Medicine, Ann Arbor, United States; 3 Michigan State University, East Lansing, United States; 4 Wayne State University, Detroit, United States; 5 University of Utah Health Care, Salt Lake City, United States; 6 Columbia University, New York, United States; 7 University of Alabama, Tuscaloosa, United States; 8 Department of Ophthalmology and Visual Sciences, University of Michigan–Ann Arbor, Ann Arbor, United States; 9 Department of Family Medicine, University of Michigan–Ann Arbor, Ann Arbor, United States

**Keywords:** Language acquisition, Medical Arabic, Arabic-speaking, Virtual curriculum, Healthcare education

## Abstract

Arabic is the sixth most spoken language in the U.S., with over 1.4 million speakers nationwide. Despite the significant population, Arabic-specific linguistic training for healthcare providers is limited. A novel Medical Arabic course was instituted to equip healthcare professionals with fundamental Arabic language skills to improve provider-patient relationships. A synchronized online course was delivered via Zoom over two semesters for three consecutive years. The course combines instructor-led lectures, interactive breakout-rooms, and role-play exercises. A post-course survey was conducted to evaluate the program’s effectiveness in improving participants’ Arabic language skills and confidence in engaging with Arabic-speaking patients. 117 participants completed Medical Arabic workshop I (MA-I) over the course of three semesters and 47 participants completed MA-II over the course of two semesters. Among 32 survey participants, 28% (*n* = 9) were graduate students in healthcare fields—including 19% (*n* = 6) medical students. 59% (*n* = 19) of respondents reported interacting with Arabic-speaking patients at least a few times a month. After completing the course, most participants felt “extremely or somewhat comfortable” using Arabic in medical context, including asking about medical history (81%, *n* = 26), communicating in a medical setting (78%, *n* = 25), recognizing medical Arabic terms (81%, *n* = 26), explaining basic diagnoses in Arabic (66%, *n* = 21), and giving physical exam instructions (56%, *n* = 18). Medical Arabic enhances healthcare providers’ comfort and confidence in using Arabic with patients, contributing to stronger communication and improved care for Arabic-speaking patients. By building language proficiency, this course helps address critical gaps in provider preparedness, ultimately promoting more equitable, patient-centered care.

**Supplementary Information:**

The online version contains supplementary material available at 10.1007/s10903-025-01793-9.

## Background

The number of Arabic speakers in the United States has steadily increased over the past 5 decades, positioning Arabic as the sixth most commonly spoken language [1]. An estimated 1.4 million individuals in the U.S. speak Arabic at home, with a significant proportion being immigrants [1]. Since 2008, Arabic-speaking refugees have been the largest group of refugees arriving in the US [2]. Despite this growing population, data on Arab-American patients within the healthcare system is limited, primarily due to the absence of Arab identifiers in healthcare documentation with “Middle Eastern and North African” only recently being included in the census as a demographic group [3]. Existing studies indicate that Arab-Americans have disproportionately higher rates of health conditions, such as metabolic syndrome and diabetes, and often lack access to health education resources [4]. Additionally, Arab-Americans face increasing rates of mental health diagnoses such as depression and anxiety [4]. These findings highlight the importance of developing targeted care strategies to address cultural and linguistic barriers.

To our knowledge, no dedicated Arabic language curriculum has been developed to support provider-patient interactions. Research has shown that language barriers significantly hinder healthcare access and quality, with Arabic-speaking immigrants reporting lower self-rated health compared to both U.S.-born Arab-Americans and English-speaking immigrants [5]. Limited English proficiency can delay access to care, hinder patient-provider communication, and negatively impact chronic disease management [6]. 

Expanding linguistic competency in healthcare has been shown to improve patient satisfaction, adherence to treatment plans, and overall health outcomes [7]. Spanish language programs for healthcare professionals improved provider comfort and ability to deliver care to Spanish speaking patients [8, 9]. Given the growing Arabic-speaking population in the United States, the implementation of structured Arabic language training for healthcare providers is a critical step in addressing disparities in healthcare delivery. To address this gap, the Medical Arabic program was developed as a novel curriculum aimed at equipping healthcare providers with basic spoken Arabic skills to better serve Arabic speaking patients.

## Theoretical/Conceptual Framework

### Course Structure

This course was introduced at the University Michigan School in October 2022 and has run for a total of five semesters. It is designed to accommodate individuals in healthcare professions with limited or no prior knowledge of the Arabic language.

#### Instructor Based Lecture

The Medical Arabic curriculum is a virtual ten-session course taught weekly over two semesters. Sessions consist of a synchronous one-hour lecture and thirty minutes of practice-based learning. The lecture portion of the course is delivered by a qualified Arabic instructor, starting with foundational skills like pronunciation and recognition of Arabic letters and diacritics. Students are introduced to basic terms and phrases, focusing on greetings and simple conversations used in medical settings. The second semester follows a similar structure with emphasis on more complex conversational skills, taking a basic patient history, and specialty-specific terminology.

#### Interactive Breakout Rooms

A unique aspect of the course is the thirty-minute breakout sessions led by native Arabic speakers proficient in both Arabic and healthcare terminology. These small group discussions allow for more personalized instruction in conversational Arabic. Students are provided with transcripts of simulated patient-provider interactions for practice. Native speakers provide direct and personalized feedback to help learners understand nuances in pronunciation, dialects, and culturally appropriate responses. Breakout rooms are organized according to learners’ self-identified proficiency levels.

### Course Material

The primary resources are the “Alif Baa” and “Al-Lisan” textbooks for beginners. Supplementary handouts, Quizlet and Kahoot were also utilized for practice. Students were required to complete graded assignments after each session. Sessions are recorded to allow enrolled students the opportunity to review material after the synchronous class.

## Methods

### Participants Data Collection

During the Fall of 2024 a survey was distributed to class participants via Canvas announcement and the Medical Arabic WhatsApp group chat and listserv. Participants who had taken the course in any prior semester including Fall 2023, Spring 2024 and Fall 2024 were eligible to take the survey.

### Measures

The survey evaluated participants’ initial and final language proficiency and confidence in working with Arabic-speaking patients (see supplement).

### Statistical Analysis

An analysis of covariance (ANCOVA) using IBM SPSS Statistics (Version 30.0) was conducted on post-course survey data (*N* = 32). ANCOVA models examined whether post-course proficiency in Arabic (speaking, reading, or writing), which were categorized in 5 levels (from low to high), significantly influenced participants’ adjusted comfort levels when using Arabic with patients, after controlling for baseline proficiency in speaking, reading, and writing.

#### Ethical Considerations

The Institutional Review Board granted an exemption for this study. All procedures involving human participants were conducted in accordance with the ethical standards of the University of Michigan IRB and the 1964 Helsinki Declaration and its later amendments or comparable ethical standards.

## Results

### Demographics

Over a span of three years, 300 students participated in the course, resulting in 164 successful course completions (Table 1). 117 participants completed Medical Arabic workshop I (MA-I) and 47 participants from those 117 who took MA-I went on and completed MA-II. Course participants included students, staff and trainees from all medical schools in the state of Michigan and at least ten additional medical schools across the US. The post-course survey was completed by a total of 32 participants (Table 2). The average age of participants was 18–24 and 69% (*n* = 22) were female. Of survey respondents, 28% (*n* = 9) identified as current graduate students in a healthcare-related field, including 19% (*n* = 6) medical students. 59% (*n* = 19) of survey respondents indicated that they interact with Arabic-speaking patients at least “a few times a month,” including 31% (*n* = 10) who interact with Arabic-speaking patients at least “a few times a week.”

**Table 1:** Number of participants who enrolled and completed medical Arabic courses from 2022 to 2025

Course name	Took the course but did not complete (*n*)	Completed successfully (*n*)	Total participated (*n*)
Medical Arabic I - Fall 2022	62	57	119
Medical Arabic I - Fall 2023	26	32	58
Medical Arabic II - WIN 2024	17	26	43
Medical Arabic I - Fall 2024	14	28	42
Medical Arabic II - WIN 2025	17	21	38

**Table 2:** Survey participant demographic data

	Count
*Education Level*	
Undergraduate Students	8
Bachelor’s Degree Holders (no further education)	9
Graduate Students (Medical Students)	9 (6)
Graduate Degree Holders	6
*Ethnic Background*	
Middle Eastern/North African	25
White/Caucasian	3
Native American/Pacific Islander	1
Other	3
*Gender*	
Male	10
Female	22

### Self-Reported Speaking, Reading and Writing Proficiency Levels

In the post-course survey, participants were asked to rank their proficiency in speaking, reading and writing before and after the course on a six-point scale, ranging from no proficiency to native proficiency. Of the participants, 25% (*n* = 8) rated their speaking proficiency as either “elementary” or “no speaking proficiency” in Arabic prior to the course. Among those with elementary or no speaking proficiency, 88% (*n* = 7) reported an improvement in their speaking proficiency level post-course. Similarly, 41% (*n* = 13) of respondents indicated “elementary reading proficiency” or “no reading proficiency” pre-course. When asked to rate their reading skills after the course, 85% (*n* = 11) of participants with initial elementary or no reading proficiency reported improvement. Additionally, 38% (*n* = 12) of survey participants noted “elementary writing proficiency” or “no writing proficiency” pre-course. Among those with no writing and elementary proficiency, 75% (*n* = 9) saw improvements in their writing skills post-course.

### Self-Reported Comfort Levels

A majority of post-course survey respondents reported feeling “extremely or somewhat comfortable” using Arabic in various medical contexts. Specifically, 78% (*n* = 25) felt comfortable communicating in Arabic in a medical setting, 81% (*n* = 26) felt comfortable asking about medical history, 81% (*n* = 26) felt comfortable recognizing medical Arabic terms related to body parts, symptoms, and diseases, 66% (*n* = 21) felt comfortable explaining basic diagnoses, and 56% (*n* = 18) felt comfortable giving physical exam instructions. Post-course speaking proficiency significantly predicted participants’ comfort in taking a medical history in Arabic, after controlling for baseline speaking proficiency (F = 3.860, *p* =.010, ηp²=0.44) (Table 3).

**Table 3:** Association between reported speaking, reading and writing proficiencies and comfort using Arabic with a patient or taking a medical history in Arabic

Model	Covariate^1^	F (Covariate)^2^	*p* (Covariate)^3^	Dependent Variable^4^	Fixed Factor^5^	F (Factor)^6^	*p* (Factor)^7^	η_*p*_² (Effect Size)^8^
**1a**	Speaking Before	0.661	0.424	**Comfort using Arabic with Patient**	Speaking After	2.262	0.079**	0.31
**1b**	Reading Before	1.704	0.203	**Comfort using Arabic with Patient**	Reading After	0.967	0.442	0.13
**1c**	Writing Before	1.141	0.296	**Comfort using Arabic with Patient**	Writing After	1.673	0.181	0.27
**2a**	Speaking Before	3.836	0.061**	**Comfort taking medical history**	Speaking After	3.860	0.010*	0.44
**2b**	Reading Before	0.260	0.438	**Comfort taking medical history**	Reading After	1.309	0.293	0.17
**2c**	Writing Before	1.751	0.199	**Comfort taking medical history**	Writing After	1.690	0.177	0.27
**3a**	Speaking Before	0.672	0.420	**Comfort explaining diagnosis**	Speaking After	1.275	0.306	0.20
**3b**	Reading Before	0.349	0.560	**Comfort explaining diagnosis**	Reading After	1.582	0.209	0.20
**3c**	Writing Before	1.168	0.291	**Comfort explaining diagnosis**	Writing After	1.974	0.121	0.30

^1^Covariate: Baseline (pre-course) self-rated proficiency in the corresponding domain, included to adjust for prior differences in skill level

^2–3^F (Covariate) and p (Covariate): F-statistics and p-values for the covariate, testing whether baseline proficiency had an independent association with post-course comfort

^4^Dependant Variable: Each model evaluates participants’ self-reported comfort performing a specific clinical task in Arabic (e.g., speaking with patients, taking a medical history, or explaining a diagnosis)

^5^Fixed Factor: Post-course self-rated proficiency in Arabic, categorized into 3 skill domains: speaking, reading, and writing. Each domain was analyzed in a separate model

^6–7^F (Factor) and p (Factor): F-statistics and corresponding p-values for the fixed factor, indicating whether post-course proficiency significantly predicted comfort after controlling for baseline proficiency

^8^η² (Effect Size): Partial eta squared values reflect the magnitude of the effect of post-course proficiency on comfort, after adjusting for baseline proficiency

0.01 = small effect

0.06 = medium effect

0.14 or higher = large effect

Most models showed large effect sizes, indicating that changes in post-course proficiency were meaningfully associated with increased comfort

*p <.05 indicates statistical significance

**p values between 0.05 and 0.10 indicate a trend toward significance

## Discussion

The findings reported here highlight the impact of a novel Medical Arabic curriculum, which has been implemented over three years to equip healthcare professionals and students with language skills essential for clinical practice. This program addresses a critical gap by providing targeted linguistic training that fosters more effective communication with Arabic-speaking patients.

A key strength of the Medical Arabic program is its practical, healthcare-focused approach, which emphasizes active learning and direct engagement with native speakers. Unlike traditional language courses, this curriculum is tailored for medical professionals, providing learners with the opportunity to practice real-world patient interactions in a structured setting. The virtual format enhances accessibility, allowing participants from various geographic locations and professional backgrounds to benefit from the training. At the time of this writing the course has served learners from at least twenty universities in the US as well as health professionals in the community who demonstrated interest in joining the course. Organizing breakout rooms based on self-reported proficiency levels enhances peer learning while maintaining the quality of instruction. Additionally, participants have the opportunity to retake the course several times to further enhance their knowledge and benefit from the different levels of breakout rooms.

### New Contribution To the Literature

Our data demonstrate that the Medical Arabic program effectively enhances both participants’ perceived language proficiency and confidence in using Arabic in medical settings. This aligns with findings from similar programs, such as a virtual Medical Spanish course that reported improved student comfort during Spanish-language patient encounters [10]. Participants with lower baseline proficiency reported substantial improvements in speaking, reading, and writing abilities, as well as increased comfort in conducting basic patient interactions. For participants with more advanced proficiency levels, the course offered an opportunity to expand vocabulary and discuss terminology-specific questions.

Future directions for the program include expanding the curriculum to offer specialized tracks for different healthcare fields, such as pharmacy or nursing. Additionally, longitudinal studies are needed to assess the sustained impact of Medical Arabic training on patient care, including measures such as patient satisfaction, adherence to treatment, and reductions in healthcare disparities. By continuing to refine and expand the program, Medical Arabic has the potential to play a critical role in bridging language gaps and improving healthcare access for Arabic-speaking communities.

### Limitations

The impact on students with prior Arabic experience is difficult to assess as their high baseline proficiency may have masked improvements in nuanced areas such as medical vocabulary and pronunciation. Self-assessment bias may have influenced the results.

## Supplementary Information

Below is the link to the electronic supplementary material.

Supplementary Material 1

## Data Availability

Raw data is provided here: https://docs.google.com/spreadsheets/d/1e1ulrGS-bkW06RVp2umwGLKw_IayPUtZ/edit? usp=sharing&ouid=107057927396141068636&rtpof=true&sd=true.
