# Supplementary material for: A Medical Arabic Course for Healthcare Professionals
Source: J Immigr Minor Health. 2025 Oct 25;28(3):762–7. doi: 10.1007/s10903-025-01793-9 (PMC13222226; doi:10.1007/s10903-025-01793-9)
Supplement: Supplementary file 1 — Supplementary Material 1 [file 10903_2025_1793_MOESM1_ESM.pdf]

[redacted]

Informed Consent

Thank you for taking part in Medical Arabic at the University of Michigan! Please take the time to fill out this pre-survey. We use this information to understand the impact of our course.

Benefits of the research: Our research aims to improve the quality of care provided to Arab Americans by addressing the cultural and linguistic barriers facing patients and providers. The goal of this session is to teach common medical terms in Arabic, introduce techniques for better communication with Arab Americans with limited English proficiency, and share common cultural practices among Arab Americans that may impact medical care. By enhancing provider

cultural competence, we hope to significantly improve the quality of care delivered to this community.

Risks and discomforts: none

Compensation: none

Participating in this study is completely voluntary. Even if you decide to participate now, you may change your mind and stop at any time. You may choose not to answer any pre-survey questions or attend or participate in the seminar, for any reason. Information collected in this project may be shared with other researchers, but we will not share any information that could identify you. If you have questions about this research study, please contact [redacted] ***or*** [redacted]

Time Expected to finish the survey: No more than 10 minutes to complete.

Study ID: HUM00230971

Demographics

Page 1/3

What is your **age**?

- 18-24
- 25-34
- 35-49
- 50 or older
- Prefer not to say

How would you describe your **gender** identity?

- Male

- Female
- Non-binary or another gender
- Prefer not to say

What is your **ethnic background**?

- White/Caucasian
- Black or African American
- Native American or Pacific Islander
- Hispanic
- Middle Eastern or North African
- Prefer not to say
- Other

What institution or school are you affiliated with?

What is your highest level of education?

- High School
- Bachelor's Degree
- Graduate Degree (MD/DO/PhD/JD/PharmD/DDS)
- Other

Have you taken the Pre-course survey?

- Yes
- No

Have you taken a Medical Arabic post-survey in the past?

- Yes
- No

Have you attended or completed any part of Medical Arabic in the past?

- Yes
- No

[IF YES ABOVE] In what semesters did you attend or complete any part of Medical Arabic? (Select all that apply)

- Fall 2022
- Fall 2023
- Spring 2024
- Fall 2024
- Spring 2025

[IF YES ABOVE] How much of Medical Arabic did you complete?

- I completed the entire class
- I attended some of the sessions
- I attended one session

Do you work in healthcare?

- Yes
- No

[IF YES ABOVE] Please specify your profession.

- Nurse
- Nurse Practitioner (NP)
- Physician
- Physician Assistant (PA)
- Dentist
- Medical/Dental/Nursing Assistant
- Pre-Health
- Pharmacist
- Medical/Dental/Pharmacy/Nursing Student
- Resident
- Fellow

- ◯ [ ] Other

In what city or zip code do you primarily work in health care, practice medicine, and/or receive your medical training?

[ ]

On average, how frequently do you work with Arabic speaking patients?

- ◯ Multiple times per day
- ◯ Once per day
- ◯ A few times per week
- ◯ A few times per month
- ◯ A few times per year
- ◯ Never
- ◯ [ ] Other

How would you rate your Arabic language **speaking** abilities **before** taking this course?

	No Proficiency	Elementary Proficiency: can form basic sentences (ask/answer simple questions)	Limited Working Proficiency: basic work commands and social phrases - limited casual conversations	Professional Working Proficiency: speak at normal speed in language - fairly extensive vocabulary	Full Professional Proficiency: advanced discussions on a wide range of topics, extensive vocabulary, can converse with ease	Native or Bilingual Proficiency: either raised speaking the language or completely fluent
Speaking	○	○	○	○	○	○

How would you rate your Arabic language **reading** and **writing** abilities **before** taking this course?

	No Proficiency: No practical ability to read or write in the language.	Elementary Proficiency: Can read and write simple phrases or sentences.	Limited Proficiency: Able to read and write straightforward texts on familiar topics but may require assistance with more complex or unfamiliar content.	Professional Working Proficiency: Able to read and write with accuracy on a range of topics, including professional and technical subjects, but not with the full idiomatic fluency or subtlety of a native speaker.	Full Professional Proficiency: Can read and write with high accuracy and fluency in professional contexts.	Native or Bilingual Proficiency: Can read and write with complete fluency, as well as understand and produce all nuances of the language at the same level as a well-educated native speaker.
Reading	○	○	○	○	○	○
Writing	○	○	○	○	○	○

How would you rate your Arabic language **speaking** abilities **after** taking this course?

	No Proficiency	Elementary Proficiency: can form basic sentences (ask/answer simple questions)	Limited Working Proficiency: basic work commands and social phrases - limited casual conversations	Professional Working Proficiency: speak at normal speed in language - fairly extensive vocabulary	Full Professional Proficiency: advanced discussions on a wide range of topics, extensive vocabulary, can converse with ease	Native or Bilingual Proficiency: either raised speaking the language or completely fluent
Speaking	○	○	○	○	○	○

How would you rate your Arabic language **reading** and **writing** abilities **after** taking this course?

	No Proficiency: No practical ability to read or write in the language.	Elementary Proficiency: Can read and write simple phrases or sentences.	Limited Proficiency: Able to read and write straightforward texts on familiar topics but may require assistance with more complex or unfamiliar content.	Professional Working Proficiency: Able to read and write with accuracy on a range of topics, including professional and technical subjects, but not with the full idiomatic fluency or subtlety of a native speaker.	Full Professional Proficiency: Can read and write with high accuracy and fluency in professional contexts.	Native or Bilingual Proficiency: Can read and write with complete fluency, as well as understand and produce all nuances of the language at the same level as a well-educated native speaker.
Reading	○	○	○	○	○	○
Writing	○	○	○	○	○	○

Please indicate your level of comfort in the following situtations.

	Extremely Uncomfortably	Somewhat Uncomfortable	Neither Comfortable nor Uncomfortable	Somewhat Comfortable	Extremely Comfortable
Using Arabic in a medical setting with patients?	○	○	○	○	○
Your ability to communicate in Arabic outside the medical context?	○	○	○	○	○
Introducing yourself in Arabic to a patient?	○	○	○	○	○
Asking basic medical history questions in Arabic to a patient?	○	○	○	○	○
Explaining basic diagnoses in Arabic to patients?	○	○	○	○	○
Providing instructions for conducting a basic physical exam in Arabic to a patient?	○	○	○	○	○

	Extremely Uncomfortably	Somewhat Uncomfortable	Neither Comfortable nor Uncomfortable	Somewhat Comfortable	Extremely Comfortable
Recognising Arabic medical terms related to body parts, symptoms, and diseases?	○	○	○	○	○

How likely are you to continue practicing the Arabic language outside of the course, now that you have completed it?

	Not likely at all	Slightly likely	Moderately likely	Very likely	Extremely likely

0 1 2 3 4 5

Click to write Choice 1

Course Design

Page 2/3

To what extent did this course improve your understanding of the cultural norms and expectations of Arabic speaking patients in the healthcare setting?

	No improvement	Slight improvement	Moderate improvement	Considerable improvement	Significant improvement
	0 1	2	3	4	5
Click to write Choice 1					

How likely are you to recommend this Medical Arabic course to a colleague in the healthcare field?

	Not likely at all	Slightly likely	Moderately likely	Very likely	Extremely likely
	0 1	2	3	4	5
Click to write Choice 1					

Rate the importance of the following to you:

	Not at all important	Slightly important	Moderately important	Important	Very Important
Learning Arabic alphabets, reading, and writing using health-related terminology.	○	○	○	○	○
Learning body parts, feelings, and expressions of pain.	○	○	○	○	○
Learning about family members, sicknesses, and diseases.	○	○	○	○	○
Learning how to check in for care, learning about days and numbers, and asking the patient about their medical concerns.	○	○	○	○	○
Learning about checking the patient's documentation, medical history, consent form, and insurance.	○	○	○	○	○

	Not at all important	Slightly important	Moderately important	Important	Very Important
Learning about taking a patient's vital signs and conducting a physical examination.	○	○	○	○	○
Learning about lab work and communicating with the caregiver or guardian.	○	○	○	○	○
Learning about Operating Room procedures and explaining outcomes.	○	○	○	○	○
Learning about prescribing medicine and giving instructions to patients.	○	○	○	○	○
Learning about Dentistry, mouth health care terminology, dental examination procedures, and medication.	○	○	○	○	○

	Not at all important	Slightly important	Moderately important	Important	Very Important
Learning about women's health terminology, procedures, and medication.	○	○	○	○	○

Please tell us (in English) the most important vocabulary words (not necessarily medical terms) that are crucial to your field that you would like to learn in Arabic.

The following questions are intended to preliminarily assess your Arabic proficiency. Please answer the questions to the best of your ability.

Arabic language is written from:

- ○ right to left
- ○ left to right
- ○ top to bottom
- ○ bottom to top

Arabic letters have upper case and lower case

- ○ True
- ○ False

The following letters connect correctly as follows: ك ت ا ب ي

- ○ ك تابي
- ○ كتبلي
- ○ كتابي
- ○ بكت لي

The word (مرحبا) means:

- Thank you!
- Hello!
- Excuse me!
- I am sorry!

Which of the following words is a feminine form of the word:

- قلمٌ
- كتابهُ
- مدرسة
- أبوهُ

How many Arabic letters are in the word يُدَرِّبُ ?

- 4
- 5
- 6
- 7

The “laam” لـ in which of the following words is silent:

- ◯ المكتب
- ◯ المدرسة
- ◯ الشمس
- ◯ القمر

The word أخت in Arabic means:

- ◯ Brother
- ◯ My aunt
- ◯ My sister
- ◯ Sister

The sentence أشعرُ بِألمِ في صدري means:

- ◯ I feel pain in my hand.
- ◯ I have an allergy.
- ◯ I need water.
- ◯ I feel a pain in my chest.

The words غرفة العمليات means:

- Waiting Room.
- Operation Room.
- Emergency Room.
- Recovery Room.

Final Thoughts

Page 3/3

What do you hope to gain from Medical Arabic?

How do you envision yourself applying the Arabic skills you acquire through the Medical Arabic course?

Do you have any suggestions for improving this course?

Please share any other thoughts, comments, and/or concerns you have about the Medical Arabic course.

Disclaimer: This is the last page of the survey. By hitting next your response will be **submitted** and recorded.
